# O.4.1-10 The FITT guidelines for multicomponent fall prevention programmes for community-dwelling older adults

**DOI:** 10.1093/eurpub/ckad133.175

**Published:** 2023-09-11

**Authors:** Katja Tomazin, Tjaša Knific, Maja Dolenc

**Affiliations:** University of Ljubljana, Faculty of Sport, Slovenia; National Institute of Public Health Slovenia; katja.tomazin@fsp.uni-lj.si; University of Ljubljana, Faculty of Sport, Slovenia

## Abstract

**Purpose:**

The most commonly proposed intervention for fall prevention in community-dwelling older adults is a multicomponent exercise programme. This is a structured form of exercise that improves muscle performance, balance, proprioception, coordination, mobility, and gait. It usually includes balance and functional exercises versus control. Although it has been suggested that exercise programmes for fall prevention should be performed three or more times per week, no precise guidelines for frequency (F), intensity (I), T(time), and T(type) of a multicomponent exercise programme have been proposed. It has already been pointed out that frequency, type, and time of a multicomponent exercise programme do not explain the effects of balance exercises in older adults. The aim of this systematic review was to evaluate the reporting of FITT guidelines for a multicomponent exercise programme for fall prevention in community-dwelling older adults, with a focus on intensity.

**Methods:**

Systematic reviews of randomised control trials comparing the effectiveness of structured, multicomponent fall prevention programmes in community-dwelling older adults were included. Studies that focused on a specific condition, such as stroke, were excluded. We included only studies that compared fall rates before and after the multicomponent programme. PubMed, PEDro, EMBASE, Scopus were searched until January 2023.

**Results:**

Preliminary results showed that FITT guidelines for multicomponent exercise were not consistently reported across studies. In particular, exercise intensity was often not reported. Without a valid and reliable measure of exercise intensity, the dose-response relationship for a multicomponent exercise programme should be further investigated.

**Conclusions:**

Limitations of this systematic review include variability in the design of multicomponent interventions and incomplete reporting of FITT in reported studies.

**Support/Funding Source:**

The study is founded from Next Generation EU Recovery and Resilience Facility and resources from National institute of public health Slovenia.

